# Microbial gatekeepers: midgut bacteria in *Aedes* mosquitoes as modulators of arboviral transmission and targets for sustainable vector control

**DOI:** 10.3389/fmicb.2025.1656709

**Published:** 2025-09-09

**Authors:** Addis Temie Worku, Andrea Sciarretta, Antonio Guarnieri, Marilina Falcone, Natashia Brancazio, Awoke Minwuyelet, Marco Alfio Cutuli, Getnet Atenafu, Daria Nicolosi, Marco Colacci, Delenasaw Yewhalaw, Roberto Di Marco, Giulio Petronio Petronio

**Affiliations:** ^1^Department of Medicine and Health Sciences “V. Tiberio”, Università degli Studi del Molise, Campobasso, Italy; ^2^Department of Agricultural, Environmental and Food Sciences, Università degli Studi del Molise, Campobasso, Italy; ^3^Department of Biology, College of Natural and Computational Sciences, Debre Markos University, Debre Markos, Ethiopia; ^4^Department of Pharmaceutical and Health Sciences, Università degli Studi di Catania, Catania, Italy; ^5^Faculty of Health Sciences, School of Medical Laboratory Sciences, Jimma University, Jimma, Ethiopia; ^6^Tropical and Infectious Diseases Research Centre, Jimma University, Jimma, Ethiopia

**Keywords:** *Aedes*, gut microbiota, symbiotic bacteria, arbovirus, vector control

## Abstract

Arboviral diseases such as Dengue virus, Zika virus, Chikungunya virus, and West Nile virus pose significant global public health and economic challenges, particularly in tropical and subtropical regions. The absence of effective vaccines and sustainable vector control strategies continues to drive high morbidity and mortality rates. Symbiotic bacteria residing in the mosquito midgut can produce antimicrobial compound, stimulate the host immune response, disrupt nutrient pathways critical for pathogen development, and interfere with the pathogen’s lifecycle and dissemination. Additionally, these microbes may reduce vector reproduction and shorten the lifespan of both immature and adult stages. Genetically modified symbiotic bacteria can release effector molecules that target pathogens without harming mosquitoes. Advances in genomic and metagenomic tools have deepened our understanding of the mosquito gut microbiome. This review highlights current knowledge of gut bacteria and arbovirus interactions and explores strategies to reduce arboviral transmission. Comprehensive literature searches were conducted using global databases, including PubMed, Web of Science, and Scopus, with a focus on English-language publications.

## 1 Introduction

Vector-borne diseases continue to pose a significant global public health challenge, particularly in tropical and subtropical regions. Despite the implementation of various intervention strategies to control these diseases, their impact remains substantial. According to the World Health Organization (2024) report, vector-borne diseases account for more than 17% of all infectious diseases and cause over 700,000 annual deaths globally. Among these, malaria alone accounts for 249 million cases and 608,000 deaths, while the remaining cases are attributed to arboviral diseases (World Health Organization, 2024).

These diseases are primarily transmitted by mosquitoes belonging to three genera: *Anopheles*, *Culex*, and *Aedes. Anopheles* mosquitoes are vectors for *Plasmodium* spp. (malaria), *Culex* primarily transmits filarial worm infections and West Nile virus (WNV). In contrast, *Aedes* species are the primary vectors of arboviruses, including Dengue virus (DENV), Zika virus (ZIKV), Chikungunya virus (CHIKV), and Yellow fever virus (YFV) (Girard et al., 2020). Arboviruses have emerged as significant public health threats due to their potential to cause explosive outbreaks and severe, sometimes life-threatening, clinical conditions (Challenges in Combating Arboviral Infections, 2024).

Among 950 *Aedes* species, *Aedes aegypti* and *Aedes albopictus* are the most efficient and widespread vectors for DENV, ZIKV, CHIKV, and YFV (De De Curcio et al., 2022; Leta et al., 2018). This is due to their adaptability to urban environments and global distribution, which contribute significantly to arboviral disease transmission. In addition, although *Aedes japonicus* is not a significant vector for arboviruses to humans, it has been collected from the field and tested positive for WNV, La Crosse, and Usutu viruses (DeCarlo et al., 2020).

Another species, *Aedes koreicus*, is native to East Asia and has recently become an invasive species in parts of Europe. It has shown potential as a vector for *Dirofilaria immitis*, *Brugia malayi*, and CHIKV (Ganassi et al., 2022). In urban areas of northern Italy, this species has been observed feeding on human blood (Montarsi et al., 2022), Further suggesting its role in arboviral transmission. *Aedes vexans* is another *Aedes* mosquito species native to Eastern Europe and a potential vector for WNV, ZIKV, and Rift Valley fever virus (RVFV) (Birnberg et al., 2019).

The primary strategies for arbovirus control rely on insecticide-based interventions, such as indoor residual spraying (IRS), space spraying, and the utilization of insecticide-treated bed nets (ITNs). However, the widespread development of insecticide resistance has significantly reduced the effectiveness of these methods (Girard et al., 2020; Minwuyelet et al., 2025). Besides chemical insecticides, vector control through habitat removal, the use of repellents, and other biological controls remain the second line of defense against arbovirus vectors. While these approaches have had some success, no single strategy has proven sufficient to control mosquito populations or eliminate arboviral transmission (Gao et al., 2020).

Considering these challenges, alternative, eco-friendly strategies are being explored. One promising avenue is the manipulation of the mosquito microbiome. Recent studies have revealed that mosquitoes harbor diverse microbiota, particularly in their gut, forming symbiotic relationships mosquito host. This microbiota can influence pathogen transmission by interacting with pathogen antagonistically or indirectly. The gut microbiota plays a crucial role in key physiological and metabolic processes in mosquitoes, including blood digestion, nutrient acquisition, reproduction, and immune modulation (Harrison et al., 2023).

The commensal and pathogenic microbiome colonization in *Aedes* mosquitos starts in early larval stages, where the aquatic environment plays a critical in shaping microbial community in midgut. During mosquito colonization, a competitive interaction occurs between commensal and pathogenic bacteria for niche establishment. While certain bacterial strains successfully establish stable symbiotic associations within specific mosquito tissues, others persist as pathogens, either causing infections in the mosquito host or exploiting the mosquito as a vector to transmit vector-borne diseases (Cai and Christophides, 2024). Microbial communities also influence development, particularly during the transition from larva to adult (Alfano et al., 2019). *Aedes* mosquito first instar larvae which grow in aseptic condition cannot survive (Coon et al., 2014). In addition, depletion of the microbiota during the larval stage significantly impairs developmental progression, leading to delayed pupation and adult emergence (Chouaia et al., 2012).

As mosquitoes transition from larvae to adults, microbial communities are maintained through transstadial transmission and environmental exposures such as sugar and blood meals. Both commensal and pathogenic microbes acquired through different feeding regimes and environmental exposure activates systemic immune responses in mosquitoes (Sharma et al., 2020). The interplay between microorganisms for nutrition and resource can modulate robust immune priming in the adult mosquito, notably through the production of antimicrobial peptides such as defensins and cecropins, regulated primarily by the Toll and IMD immune pathways (Cirimotich et al., 2011a).

In *Anopheles* mosquito gut microbiota induce systemic immunological response that limit the abundance and distribution of microorganism, and RNAi-mediated silencing of AMPs and immune signaling pathways has been shown to result in increased proliferation of the gut microbiota (Dong et al., 2009; Clayton et al., 2014). Similarly in Aedes mosquito proliferation of microbiota following blood meal activate IMD pathway and limits sindbis virus infection (Barletta et al., 2017). Moreover, studies show that certain bacteria in mosquito gut can either enhance or inhibit infections, depending on their interactions with both the pathogen and host immunity (Boissière et al., 2012; Ramirez et al., 2014; Wu et al., 2019).

Recent scientific advancements offer a novel approach to address this long-standing problem by harnessing the potential of gut microbiome in Aedes mosquitoes. A promising technique involves modifying the gut microbiome of mosquitoes to diminish their ability to transmit viruses, which are responsible for arboviral diseases (Dickson et al., 2018; Gao et al., 2020; Hegde et al., 2015).

This review synthesizes current research on the composition and factors related to the gut bacteria of *Aedes* mosquitoes, revealing its role in influencing arboviral transmission dynamics and evaluating emerging strategies using microbial communities for sustainable vector control. By integrating insights into microbiota-pathogen interactions and innovative interventions, the review aims to bridge gaps in understanding how microbial manipulation can disrupt arboviral spread and address insecticide resistance, ultimately informing next-generation, eco-friendly interventions for global arboviral disease mitigation.

## 2 Methodology

**Figure 1:**
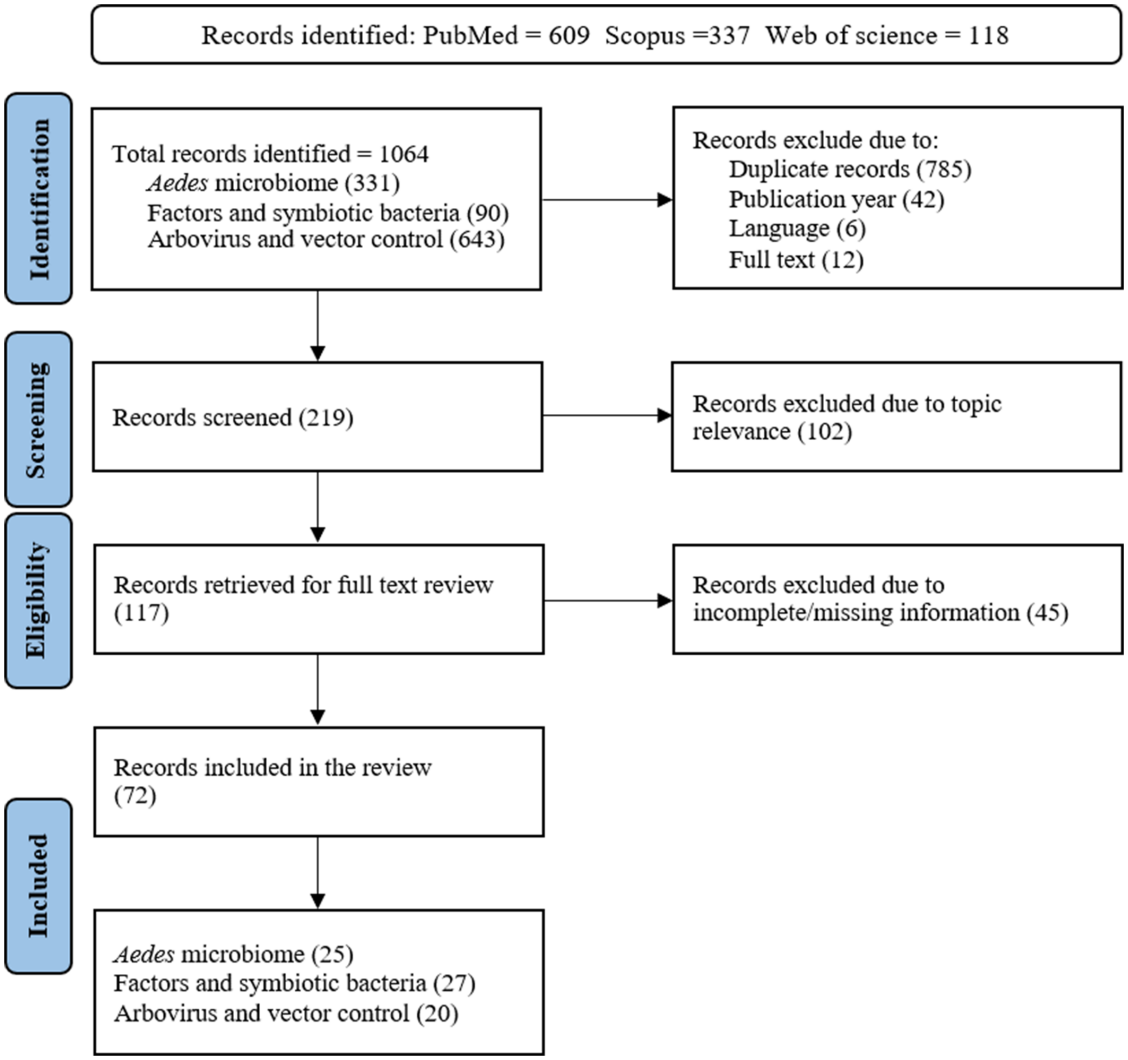
The flowchart of search and selection of articles for review of *Aedes* mosquito microbiota, factors and role in modulating arboviral transmission and vector control.

The literature search focused on primary articles, published between 2010 and 2025. The review research covered topics related to the bacterial composition of *Aedes* mosquitoes, factors influencing bacterial diversity, interactions between *Aedes* gut symbiotic bacteria and arboviruses, and their potential role in vector control. Articles were identified using Boolean operators “AND” “OR” and “NOT” in the search strategies. Key words such as *Aedes* gut microbiota, symbiotic bacteria, arbovirus, and vector control were used either separately or in combination. Studies were excluded if they focused on mosquito vectors other than *Aedes* mosquitoes, examined non-bacterial components of microbiome, or lacked clear methodologies for bacterial identification. Relevant articles published in English were identified using databases such as PubMed, Web of Science, and Scopus. The final search was conducted between January 30 and February 15, 2025. Data were extracted by analyzing the text, figures, and tables from the included articles. In this review, after examining 219 primary articles, we retrieved 72 articles (see Figure 1).

## 3 Gut microbiota of *Aedes* mosquito

### 3.1 Acquisition of gut microbiota in *Aedes* mosquito

The mosquito microbiome comprises a diverse community of bacteria, fungi, and insect-specific viruses that reside within and may spread through various mosquito tissues (Pascar et al., 2023; Guégan et al., 2018). While the majority of these microorganisms are found within the gut, they are also found in other somatic and germline tissue such as the salivary gland, crop, reproductive tract and cuticle of *Aedes* mosquitoes (Onyango et al., 2021; Valiente Moro et al., 2013).

**Figure 2:**
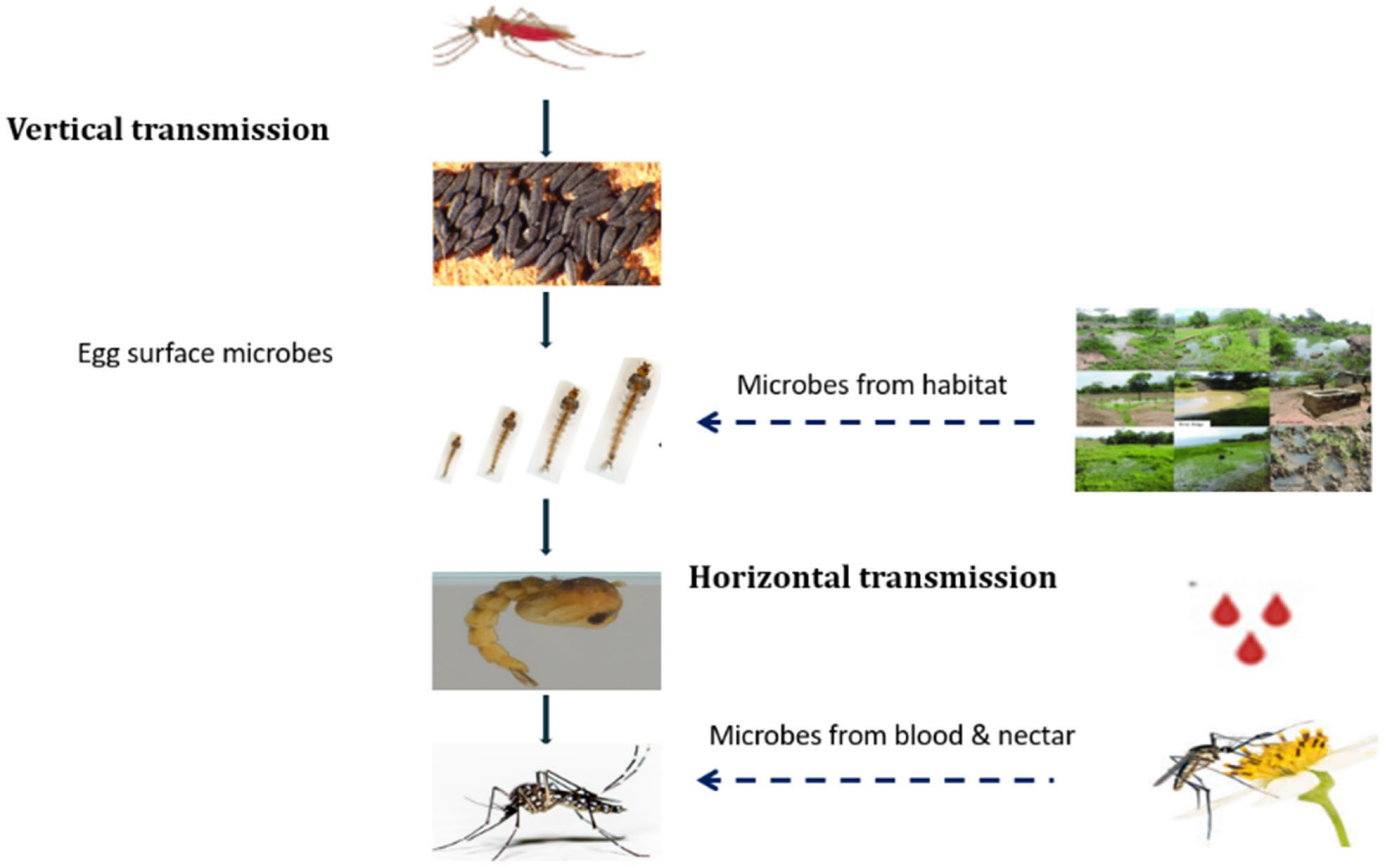
Summary of gut microbiota acquisition through vertical and horizontal transmission.

Mosquitoes can acquire their microbiota vertically from their parents. Various species of mosquitoes can vertically transmit intracellular bacteria, such as *Wolbachia,* from one generation to the next (Caragata et al., 2022). In contrast, several studies reported that microbiota are also acquired horizontally from the surrounding environment including aquatic habitat and feeding sources. Additionally, some microbial communities are transmitted via the egg surface (Coon et al., 2016). Upon hatching, first instar larvae ingest fragments of the eggshell, thereby acquiring microbes from the egg-associated microbiome (Gimonneau et al., 2014; Figure 2).

### 3.2 Composition of bacteria in *Aedes* mosquito

Bacteria represent the primary components of the mosquito gut microbiota, followed by fungi, algae, and viruses to a lesser extent (Guégan et al., 2018; Cansado-Utrilla et al., 2021). We identified twenty-five articles that focused on the bacterial composition of *Aedes* mosquito vectors. The articles included in this study used both culture-dependent and culture-independent methods. Two studies employed culture-dependent techniques, while the others utilized culture-independent approaches based on current molecular strategies such as 16S rRNA gene sequencing and metagenomic analysis, which have become essential tools for characterizing the bacterial microbiota in the mosquito gut.

Both culture dependent and culture independent studies confirmed that *Aedes* mosquitoes harbor a wide range of both classified and unclassified bacterial taxa associated with the gut (Bennett et al., 2019; Baltar et al., 2023). Among these, the most prevalent bacterial phyla identified in *Aedes* mosquitoes include Proteobacteria, Firmicutes, Actinobacteria, and Bacteroidetes (Muturi et al., 2021a; Pascar et al., 2023).

Several studies conducted in the United States, consistently reported Proteobacteria as the dominant and highly diverse bacterial phylum in *Aedes* mosquitoes. Proteobacteria were a highly diverse and dominant phylum in both the midgut and saliva of *Aedes* mosquitoes (Pascar et al., 2023; Onyango et al., 2021). Similarly, Proteobacteria remained dominant phyla across the mosquito population regardless of variation in their aquatic habitats and blood meal sources (Caragata et al., 2022; Muturi et al., 2018).

The pattern was consistent with findings from India, where Proteobacteria as the dominant phylum in the gut of *Ae. aegypti* (Yadav et al., 2016; Sarma et al., 2022). Additional studies from diverse locations, including Brazil, Korea, and China, have corroborated the widespread dominance of Proteobacteria in the gut of *Aedes* mosquitoes (Akintola and Hwang, 2024; Baltar et al., 2023; Lee et al., 2020). This widespread dominance may result either from the insect host’s active recruitment of these bacteria that support its growth or from the greater ability of Proteobacteria to invade and proliferate within new insect hosts compared to other bacterial groups.

One of the most extensively studied genera within the Proteobacteria phylum is *Wolbachia*, a maternally inherited endosymbiont with critical implications for mosquito biology and vector competence. Studies have consistently reported *Wolbachia* as an abundant midgut bacterium in *Aedes* mosquito although the proportion and prevalence vary study and may depend on environmental, geographic, or methodological factors. A study from Spain and São Tomé found *Wolbachia* to be overwhelmingly dominant with 92.4–98. 8% in Sao Tome and 96.1–97.5% in Spanish samples, with 77.22% of mosquitoes co-infected with both *w*AlbA and *w*AlbB strains (Melo et al., 2024).

A similar study from Mexico, showed that *Wolbachia* accounted for 9.6% of 16S gene sequences, with the abundance 0 to 32% in each sample. A high prevalence of the *w*AlbB strain, and included genes linked to Cytoplasmic Incompatibility (CI) was detected (Hernández et al., 2024). Molecular approaches using *Wolbachia* specific primer and strain specific genetic marker essential for strain specific comparison and identification of genes related to CI.

Species and tissue specific occurrence of *Wolbachia* has been reported in different studies. A metagenomic analysis reported that the Prevalence of *Wolbachia* was 100% in *Ae. albopictus* and *Cx. pipiens* but not in other *Aedes* or *Anopheles* species. In addition to this *Wolbachia* was found to be more abundant in reproductive tissues where as *Asaia* was predominantly detected in the gut (Ilbeigi Khamseh Nejad et al., 2024). Similar study from Southern China reported that *Wolbachia* was more abundant in the whole body of *Ae. albopictus* than in the midgut. Additionally, microbiota network analysis revealed *Wolbachia* have both positive and negative co-occurrences with other bacterial genera (Lin et al., 2021). For example, *Wolbachia* and *Asaia* shows mutual exclusion in species and host tissue level (Rossi et al., 2015). This phenomenon has important implications for symbiont-based vector control strategies.

Studies from South Korea and Malaysia reported that *Wolbachia* was the most dominant genus, accounting for 98.36% of the midguts of *Ae. albopictus* with relative abundance in female and 70.5% of the bacterial community in the midgut of *Ae. albopictus* mosquitoes with relative abundance higher in male, respectively, (Lee et al., 2020; Ilbeigi Khamseh Nejad et al., 2024). Similar study from Brazil observed higher detection rates of *Wolbachia* in field-collected mosquitoes, particularly during the dry season (Baltar et al., 2023).

Likewise, a study in southern Thailand also reported *Wolbachia* prevalent in both sexes of *Ae. albopictus*, with greater abundance in males (Rodpai et al., 2023). Sex-based microbiota profiling, sample pooling, mosquito collection season, and geographical location might cause variation to abundance. Furthermore, due to methodological differences, *Wolbachia* is an intracellular bacterium that is not grown in artificial media and detected in culture-dependent studies (see Supplementary Table 1).

Another important bacterial genus in the *Aedes* gut microbiota is *Enterobacter.* The presence of *Enterobacter* was detected in the gut of adult *Aedes* mosquitoes collected from field but not in the egg or larval stages (Hernández et al., 2024). Contrastingly, study from Italy reported *Enterobacter* was detected in both *Bacillus thuringiensis israelensis* (*Bti*) exposed adults and larvae *of Ae. albopictus*, with a significantly higher abundance in *Bti*-resistant larvae (Bahrami et al., 2024). Similarly, study from Brazil reported a 3%, prevalence of *Enterobacter* isolated only from the eggs of *Ae. aegypti* mosquitoes that obtained from laboratory colony (Gusmão et al., 2010).

The variation in *Enterobacter* abundance across different developmental stages may be attributed to differences in sample sources and exposure to *Bti* larvicide, as *Enterobacter* has been previously associated with increased insecticide resistance. Meanwhile, a study from southern China reported that *Enterobacter* was present in both the entire body and midgut of both *Aedes* mosquitoes. In terms of abundance, it was more prevalent in the midgut of female *Ae. albopictus* than in its whole body (Lin et al., 2021). Additionally, a study from Thailand found that *Enterobacter* was present in all groups tested for CHIKV infection; however, its presence did not significantly correlate with infection status (Siriyasatien et al., 2024).

A study from the USA has shown that *Enterobacter* was the dominant genus among the five found in *Ae. aegypti* mosquitoes that fed on different blood meal sources (Muturi et al., 2021a). On the other hand, studies from India and Madagascar reported that *Enterobacter* was the second most dominant bacterium isolated in both sugar-fed female and male mosquitoes (Valiente Moro et al., 2013; Yadav et al., 2016). Furthermore, studies from Thailand and India reported that species like *Enterobacter cloacae* were particularly dominant in both *Ae. aegypti* and *Ae. albopictus* across field and lab populations (Yadav et al., 2015; Tuanudom et al., 2021). *Enterobacter* is symbiotic bacteria commonly detected in the gut of *Aedes* mosquito regardless of host species, method of isolation, and diet. This promotes microbial stability through beneficial co-occurrences in mosquito guts.

The genus *Asaia*, another member of the Proteobacteria, also plays a crucial role in the microbiota of *Aedes.* Studies from Iran detected *Asaia* in the midgut of field collected *Ae. albopictus* (Darbandsari et al., 2025). Roman et al. demonstrated that *Asaia* can accelerate the growth of *Ae. aegypti* larval development and interact with the broader larval microbiome (Roman et al., 2024). Interestingly, study from Thailand, found *Asaia* in CHIKV negative and control groups, but not found in infected mosquitoes (Siriyasatien et al., 2024). Similar study from the USA also reported variable *Asaia* spp. presence in *Ae. aegypti* populations with differing DENV susceptibility, although the role of *Asaia* spp. in antiviral defense remained unclear (Chen et al., 2023). The observed difference between infected, and uninfected groups mosquitoes can a possible association implying that *Asaia* may play a protective or modulatory role in vector competence. Further experimental infection studies are important to elucidate the association. *Asaia* was the most abundant genus in the *Ae. aegypti* sample that had been treated with a blood meal containing Amox/Clav and was reported as resistant to it (Van Garcia et al., 2024). It was found in *Aedes*, *Anopheles*, and *Culex* species, with varying prevalence depending on geographical location and mosquito species (Ilbeigi Khamseh Nejad et al., 2024). In *Ae. aegypti Asaia* was abundant in the crop than in the midgut (Villegas et al., 2023). Its abundance across has been reported at low and fluctuating levels across the regions such as Italy, Spain, and São Tomé (Ilbeigi Khamseh Nejad et al., 2024; Melo et al., 2024).

Other bacterial genera within Proteobacteria frequently detected in *Aedes* mosquitoes include *Pseudomonas*, *Serratia*, *Pantoea*, *Klebsiella*, and *Aeromonas*, as reported by multiple studies across the globe (Brettell et al., 2025; Darbandsari et al., 2025; Pascar et al., 2023; Muturi et al., 2021a; Rosso et al., 2018; Minard et al., 2015).

Firmicutes represent the second most abundant phylum in many studies. A study from the USA reported that Firmicutes accounted for 36.6% of *Ae. aegypti* microbiota, *Bacillus* and *Clostridium* were found in the midgut with *Bacillus subtilis* being the most dominant species at 42.4% (Pascar et al., 2023). Similar finding was reported in China *Bacillus* and *Clostridium* were present in both *Bti*-resistant and control larvae, with *Bacillus* being the predominant genus (Bahrami et al., 2024). Firmicutes were also the second most abundant phylum (27.2%) in whole-body microbiota of *Ae. albopictus*, with *Bacillus* dominating (22.9%). In contrast, tissue specific comparative analysis showed Bacteroidetes as the second most prevalent phylum, indicating variation in microbial composition across different tissues(Lin et al., 2021).

In contrast, Actinobacteria was the second most dominant phylum (11.3%), followed by Firmicutes (10.3%), Bacteroidetes (5%) and Cyanobacteria (1.3%) in *Ae. aegypti*. In this study *Bacillus*, *Lysinibacillus*, and *Clostridium* as common genera detected in adult (Hernández et al., 2024). Similarly high levels of Actinobacteria were detected in both laboratory-reared and field-collected *Ae. albopictus* (Tuanudom et al., 2021). Acinetobacter consisted of 17% of *Ae. albopictus* bacterial community, while Bacteroidetes was the least represented phylum, characterized by a single species, *Chryseobacterium rhizoplanae*, isolated from blood-fed individuals (Yadav et al., 2016).

Actinobacteria and Firmicutes were commonly found in larvae and breeding sites, however the mosquito gut appears more selective toward these bacterial groups. For example, *Staphylococcus*, *Bacillus*, and *Clostridium* are more likely associated with hindgut or body surface than midgut lumen (Ngo et al., 2016). Under laboratory condition larvae fed controlled larval diet, organic matter is limited, Firmicutes are less supported, whereas Actinobacteria tend to persist and adapt well to these stable, low-diversity microbiota environments.

Bacteroidetes were present in lower abundance in most studies, but its enrichment in mosquito gut associated with bloodmeal, *Elizabethkingia* with a dominant genera (Sharma et al., 2020). Variation in microbial abundance between species and across geographic regions has also been reported by Pascar et al. (2023). Bacteroidetes were 4.7 and 1.5% of Actinobacteria in *Ae. aegypti* mosquitoes. Actinobacteria and Bacteroidetes were present in *Ae. albopictus* mosquitoes in low abundance, but their abundance was high in *Culex* mosquitoes (Akintola and Hwang, 2024).

Similarly, Bacteroidetes were detected in both the midgut and saliva, bacteria belonging to the genus *Elizabethkingia* were enriched in ZIKV-infected midguts. In contrast, *Wolbachia* was abundant in non-infected midguts (Onyango et al., 2024). *Elizabethkingia* enrichment in infected mosquito midguts suggests a host-pathogen interaction, potentially involving an antiviral mechanism that influences viral replication. While *Wolbachia* prevalence in uninfected mosquitoes associates with mosquito immunity and suppressing arbovirus infection and replication.

In addition to the factors related to the abundance of certain bacteria in mosquito characterization of bacteria could biased by the techniques used for studying microbiota such as DNA extraction method, primer selection, sequencing platform and bioinformatics pipeline. Level of variability within the 16S rRNA genes also making it difficult to distinguish them in species or strain level. This might cause underestimation or over estimation of certain bacteria (see Supplementary Table 1).

### 3.3 Factors that shape gut microbiota of *Aedes* mosquito

A total of twenty-seven articles were retrieved that examined the various factors influencing the mosquito microbiome. Recent studies have shown that the microbial communities of *Aedes* mosquitoes vary significantly depending on several intrinsic and extrinsic factors, including mosquito species, developmental stage, sex, larval diet, and the environment of the breeding site.

For instance MacLeod et al. (2021) found that adult mosquitoes emerging from larvae reared on a nutrient-rich diet exhibited a significantly higher bacterial load in both their midguts and breeding water. Specifically, increased dietary abundance was associated with elevated levels of *Enterobacteriaceae* and *Flavobacteriaceae* and a decrease in *Sphingomonadaceae*. Larval nutrition not only affects growth and development but also influences microbial colonization. A significant increase in *Enterobacteriaceae* in larvae-fed pelleted diets however, *Flavobacteriaceae* levels remained essentially unchanged (Linenberg et al., 2016).

Martinson and Strand (2021) showed that larvae fed a complete bacterial community alongside nutrient-rich food exhibited distinct microbial profiles. Similarly, variation in midgut bacterial communities across developmental stages, sexes, and feeding conditions has been reported. For example, *Acinetobacter pitti* was abundant in sugar-fed females and larvae, while *Pseudomonas monteilii* dominated in blood-fed mosquitoes. *Pantoea* was prominent in adult males, whereas *Chryseobacterium rhizoplanae*, the only Bacteroidetes species isolated, was found exclusively in blood-fed *Ae. albopictus* (Yadav et al., 2016).

Environmental exposure during larval or adult stage also plays a significant role in diversity of microbiota. Scolari et al. found that over 60% of the bacterial genera was conserved in both larval and adult *Ae. albopictus* were also present in breeding site water (Scolari et al., 2021). Similarly, Alfano et al. (2019) reported that 84% of the bacterial communities in the mosquito gut were varied across breeding sites, larvae, pupae, and adults, with notable shifts in dominant taxa from the larval to adult stages.

Juma et al. (2021) observed that larval sampling environments significantly influenced microbial communities in *Ae. triseriatus* and *Ae. japonicus,* with *Dysgonomonas* being the dominant genus in *Ae. triseriatus*, while *Mycobacterium* and *Carnobacterium* were dominant in *Ae. Japonicaus.* Unclassified *Comamonadaceae* was dominant in water samples (Rodpai et al., 2023) confirmed that the composition of microbiota varies significantly across developmental stages and between *Ae. aegypti* and *Ae. albopictus.* While transstadial transmission of microbiota was observed, adult mosquitoes showed a reduced bacterial load compared to larvae. Microbiota also varies species to species, *Wolbachia* was more abundant in *Ae. albopictus*, whereas *Blautia* was enriched in *Ae. aegypti*.

Blood feeding has a profound effect on gut microbiota. Sarma et al. (2022) demonstrated a significant difference in the gut microbiota of *Ae. aegypti* depending on feeding status: *Rhodobacterales* and *Neisseriales* were enriched in mosquitoes fed with human blood, while *Caulobacterales* dominated in unfed mosquitoes. Supporting this finding, Muturi et al. (2021a) reported that the blood source influenced the composition of midgut microbiota. For example, newly emerged adults and those fed on chicken, rabbit, and human blood were characterized by *Leucobacter*, *Chryseobacterium*, *Elizabethkingia*, and *Serratia*, respectively, Whereas sugar-fed mosquitoes harbored more *Pseudomonas*.

Salgado et al. (2024) reported lower microbiota diversity in blood-fed mosquitoes compared to sugar-fed ones, with blood digestion dominated by *Enterobacterales*, followed by a rise in *Elizabethkingia anopheles* post-digestion. LaReau et al. (2023) highlighted taxonomic and functional differences between axenic mosquitoes colonized by environmental bacteria and those reared in insectaries. The former showed greater diversity and dynamic shifts during blood feeding and could even perform hemolysis in culture.

The composition and diversity of microbial communities in both larvae and adult mosquitoes are influenced by the colonization of microorganisms. Frankel-Bricker et al. (2020) demonstrated that the fungal colonization of the gut by *Zancudomyces culisetae* in larvae reduced microbial diversity in adults and affected the transmission of specific bacterial genera. Similarly, (Yin et al., 2025) demonstrated that inoculation with *Escherichia coli*, *Staphylococcus aureus,* and *Beauveria bassiana* altered the midgut microbiota across different stages.

Wei et al. (2017) reported that *B. bassiana* infection in mosquitoes induced gut dysbiosis, increasing bacterial load while reducing diversity. The gut became dominated by *Acinetobacter*, *Serratia* and *Asaia*, with *Serratia marcescens* overgrowth leading to translocation into the hemocoel and increased mortality. *Wolbachia* infection in *Ae. aegypti* also caused microbiome shifts and negatively interacted with other taxa (Pascar et al., 2023). Notably, *Serratia* was enriched in *Wolbachia*-infected mosquitoes, while *Pseudomonas* and *Acinetobacter* dominated in *Wolbachia*-free individuals (Balaji et al., 2021).

Viral infections also influence gut microbiota. DENV infection modulates bacterial abundance in *Ae. aegypti,* upregulating *Desulfovibrionaceae* and *Enterococcus gallinarum* while reducing overall bacterial load (Zhao et al., 2022). Similarly, (Ramirez et al., 2012) showed that DENV infection significantly decreases the overall bacterial load in the midgut of *Ae. aegypti* mosquito.

In addition to viral infections, chemical insecticides also significantly alter the microbiota of mosquitoes. Arévalo-Cortés et al. (2020) observed reduced gut diversity in both ZIKV-infected and lambda-cyhalothrin–resistant mosquitoes. *Bacteroides vulgatus* were enriched in ZIKV-infected groups, while *Pseudomonas viridiflava* and *Clostridium ramosum* were found in resistant mosquitoes. Additionally, Wei et al. (2023) demonstrated that pyrethroid exposure resulted in microbial enrichment or depletion, with genera such as *Butyricimonas, Prevotellaceae, Anaerococcus,* and *Pseudorhodobacter* significantly reduced.

Resistance mechanisms also drive microbiota shifts. *Ae. aegypti* resistant to permethrin showed different gut microbiota compared to susceptible strains (Muturi et al., 2021a). Viafara-Campo et al. (2025) found that deltamethrin-resistant females and temephos-treated larvae had distinct microbiota, with *Enterobacter* predominant in untreated females and resistant larvae, *Bacillus* exclusive to larvae, and *Serratia*, *Cedecea neteri*, and *Elizabethkingia* exclusive to resistant females. Sun et al. (2024b) similarly reported a higher abundance of certain gut symbiotic bacteria in deltamethrin field-resistant adults compared to sensitive adults; however, both field-resistant and field-sensitive adult mosquitoes exhibited significantly reduced gut microbiota diversity compared to laboratory-sensitive adults.

Antibiotic exposure also alters gut microbiota. A study by Qing et al. (2020) reported that ampicillin exposure in *Ae.albopictus* across developmental stages caused gut dysbiosis, particularly in adult females. Van Garcia et al. (2024) demonstrated that the ingestion of antibiotics during blood meals reduced microbial diversity, particularly in field-collected mosquitoes. Co-exposure with DENV-modified bacterial composition: *Pseudomonas* and *Asaia* decreased, while *Enterobacter* increased. Minard et al. (2015) also observed that larval antibiotic exposure led to a reduction in *Elizabethkingia*, elimination of *Chryseobacterium* and an increase in *Wolbachia* in adults.

Environmental pollutants, such as polycyclic aromatic hydrocarbons (PAHs), can also impact gut microbiota (Antonelli et al., 2024) reported stage-specific effects of chronic PAH exposure in *Ae. albopictus* with a greater impact on larvae. PAH exposure enriched bacterial families capable of PAH degradation, altering competitive dynamics in the gut. Moreover, CRISPR/Cas9 mediated deletion of bacteria ompA genes impaired colonization capability (Hegde et al., 2019).

Geographic distribution and environment also influence the composition of microbiota. Minard et al. (2015) found that mosquitoes invading new geographic areas had reduced microbial diversity compared to those from native regions. Brettell et al. (2025) showed that *Ae. aegypti* reared in different insectaries from eggs laid at the same time exhibited significantly different gut microbiota despite similar development.

**Figure 3:**
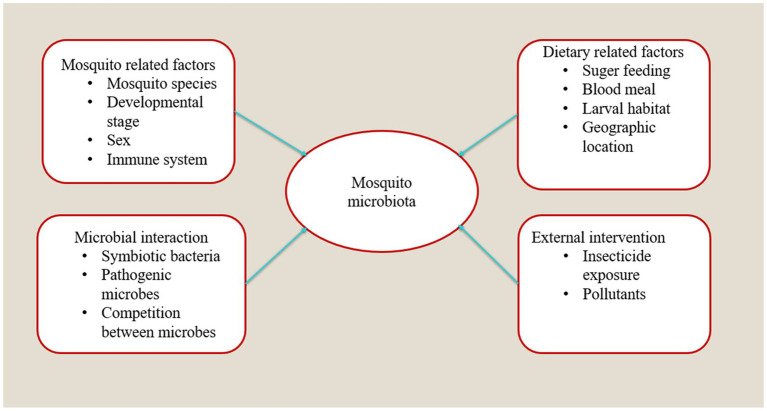
Factors that shape the gut microbiome composition of *Aedes* mosquitoes.

Similarly, *Ae.albopictus* collected from Spain and São Tomé shared core microbiota but had location-specific genera, including different *Wolbachia* strains (Melo et al., 2024). Baltar et al. (2023) observed differences between lab colonies and field-collected mosquitoes, with gut microbiota diversity decreasing from wet to dry seasons (see Figure 3; Supplementary Table 2).

### 3.4 Role of mosquitoes’ guts microbiota in modulating pathogen transmission

During blood feeding, mosquitoes might ingest pathogens, particularly *Plasmodium* parasites and/or arboviruses, which first enter the mosquito’s midgut. These pathogens penetrate the midgut epithelial cells, spread into the hemocoel, and ultimately cross the salivary gland barrier, gaining access to the saliva for transmission during subsequent bites (Mueller et al., 2010). Vector competence refers to the intrinsic ability of a mosquito to acquire, maintain, and transmit pathogens to another host. This is a complex biological trait influenced by various intrinsic and extrinsic factors, including the mosquito’s genetics and associated microbiota. The interaction between the mosquito genotype and its microbiota plays a crucial role in modulating vector competence (Cansado-Utrilla et al., 2021). Sixteen recent articles addressed the role of mosquito microbiota in pathogen transmission.

Studies have shown that the presence of a certain bacteria particularly a member of *Rickettsiaceae*, *Enterobacteriacea*e, and *Flavobacteriaceae* family can be corelated with reduced arboviral infection in mosquitoes(Kukutla et al., 2014; Apte-Deshpande et al., 2012; Moreira et al., 2009). The underlying mechanism by which the gut microbiome in *aedes* mosquito is not fully understood, but they are believed to involve both direct and indirect interactions.

Gut microbiota in mosquitoes can modify the gut environment by secreting antiviral metabolites and modulating the mosquito’s immune response, thereby inhibiting arbovirus entry, replication, and transmission. For example, *Rosenbergiella* YN46, found in field-collected *Ae. albopictus* has been shown to colonies the mosquito gut consistently. This bacterium secretes glucose dehydrogenase (RyGDH) enzyme, which changes glucose to gluconic acid during blood digestion. The accumulation gluconic acid in mosquito gut change the gut lumen to acidified environment which inactivate viruses and significantly inhibits invasion of DENV and ZIKV gut epithelial cells (Zhang et al., 2024) Similarly, *Enterobacter hormaechei* B17 (Eh_B17), a symbiotic gut bacterium, consistently colonizes the midgut of female mosquitoes after transplantation. Eh_B17 produces metabolite sphingosine, which significantly inhibits the early stages of DENV and ZIKV entry into host cells (Sun et al., 2024a).

Symbiotic bacteria can inhibit pathogen transmission by computing pathogen essential resources that are important for growth, replication and transmission. *Wolbachia,* an intracellular symbiotic bacterium, is widely used in mosquito control strategies and demonstrates antiviral properties. Transient somatic infections with *Wolbachia* strains *w*Alb and *w*Mel significantly reduced Mayaro virus (MAYV) infection and viral titters in a strain-specific fashion. However, *Wolbachia* causes enhancement to Sindbis virus infection (Dodson et al., 2024). *Wolbachia,* alters cholesterol metabolism by diverting host resources from the mevalonate (MVA) pathway and downregulating cholesterol esterase genes, which are typically upregulated during ZIKV infection. This metabolic disruption depletes lipid droplets and inhibits ZIKV replication within mosquito cells (Edwards et al., 2023).

*Wolbachia* infection primarily blocks virus transmission, the mechanism is not fully explored yet, it could be activating the mosquito’s innate immune system or outcompeting with intracellular resource. *Wolbachia* strain NC-*w*Mel, derived from crosses between Australian *w*Mel females and New Caledonian wild-type males, and *w*Mel-Sg from Singapore significantly reduced susceptibility to and blocked transmission of ZIKV, DENV, and CHIKV in *Ae. aegypti.* Notably, mosquitoes infected with NC-*w*Mel exhibited complete CI and efficient maternal transmission (Pocquet et al., 2021; Tan et al., 2017). Similarly, populations of *Wolbachia*-infected *Ae. aegypti* (wMel), both in the field and in the laboratory, showed a significant reduction in DENV transmission potential and experienced an extended extrinsic incubation period of 4–7 days (Carrington et al., 2018).

Mosquito associated symbiotic bacteria also modify arboviral transmission by altering the expression or function of conserved mosquito proteins required for viral entry, replication and attachment. For example, *Wolbachia* (*w*AlbB) inhibits DENV-2 replication, Aag-2 Cells. *w*AlbB inhibited virus genome replication by blocking synthesis of the viral negative-strand RNA. In addition to this *w*AlbB inhibit DENV binding to Aag-2 cells by downregulating transcription of host membrane binding protein dystroglycan and beta-tubulin (Lu et al., 2020). Pelo protein is a conserved protein in insects involved in immune regulation, promoting *Drosophila* C virus replication in *D. melanogaster* (Wu et al., 2014). *Wolbachia-*infected *Ae. aegypti* females (*w*MelPop-CLA), showed reduced expression of Pelo and altered subcellular localization, which could potentially contribute to decreased DENV replication (Asad et al., 2018).

Symbiotic bacteria in the mosquito gut can produce natural toxins, antiviral compounds, or metabolites that prevent viruses from attaching to the gut lining and promote the degradation of viral genomes before attachment. The previous ingestion of *Chromobacterium* sp. Panama (Csp_P) by mosquitoes significantly reduced susceptibility to *P. falciparum* and DENV infection, both *in vitro* and *in vivo* (Ramirez et al., 2014). In support of this, *Csp_*Panama exhibits an inhibitory effect on DENV replication both in mosquitoes and *in-vitro*. Neutral protease and amino-peptidase enzymes destabilize the virus by degrading the viral envelope protein. This degradation of the viral envelope protein inhibits viral attachment to the host cell (Saraiva et al., 2018).

Likewise*, Chromobacterium* sp. Beijing *(Csp_BJ),* isolated from *Ae. aegypti* produces two antiviral effectors, CbAE-1 and CbAE-2, with conserved lipase domains. These lipases disrupt viral envelopes, thereby inactivating DENV, *Japanese encephalitis* virus (JEV), YFV, and ZIKV. Furthermore, high doses of *Csp*_BJ administered orally result in significant mortality in mosquitoes (Yu et al., 2022). Prostaglandins (PGs), immune-active lipids, are produced by midgut tissues in response to microbiota and play crucial roles in mosquito immunity. *Enterobacter cloacae* triggers PG production in the midgut of *Ae. aegypti* and in *Aag2* cells, which in turn enhance antiviral immune responses against DENV (Barletta et al., 2020).

Introduction of symbiotic bacteria isolated from mosquito guts of antibiotic-treated mosquito shows a significant role in modulating viral replication.,by boosting mosquitoes innate immune system, particularly the upregulation of AMPs, and upregulation of immune pathway leading to reduced viral infection and viral titters. Furthermore, microbial competition between symbiont and viruses in the gut creates a hostile environment for viral replication. *Proteus* sp. and *Paenibacillus* sp. were introduced through blood meals and significantly reduced DENV infection and viral titters in aseptic mosquitoes. Notably, sugar meal supplementation with *Proteus* spp. also decreased DENV infection rates (Ramirez et al., 2012).

Similarly, *Elizabethkingia anopheles aegypti* colonize *Ae, albopictus* resulted in lower average ZIKV infections and reduced viral loads in Vero cell assays for ZIKV, DENV, or CHIKV (Onyango et al., 2021). *Lysinibacillus* spp., previously recognized for its larvicidal activity, was recently shown to reduce ZIKV viral loads in the head and thorax of *Ae. aegypti*, with no detectable virus in the saliva following forced feeding (Do Nascimento et al., 2022; see Table 1).

**Table 1:** Summaries of the role of mosquitoes’ gut microbiota in pathogen transmission prevention.

Mosquito species	Bacterial strain	Pathogen	Method *in vivo*/*in vitro*/cell line	Experimental findings	References
*Ae. albopictus* & *Ae. aegypti*	*Rosenbergiella_*YN46	DENV and ZIKV	*In vivo* feeding bacteria to mosquitoes & *In vitro*	Inhibiting gut epithelium infection	Zhang et al. (2024)
*Ae. aegypti* and *Ae. albopictus*	*Enterobacter hormaechei*_B17(Eh_B17)	DENV & ZIKV	*In vivo* mosquito feeding & *In vitro* C3/C6 cell line & Vero cell	Block viral infection entry stage of virus	Sun et al. (2024b)
*Ae. aegypti*	*Chromobacterium* sp. Panama (Csp_P)	DENV	*In vitro* cell line	Secret protein that degrades viral envelope protein prevents viral attachment	Saraiva et al. (2018)
*Ae. aegypti*	*Chromobacterium* sp. Beijing (Csp_BJ)	DENV, ZIKV, JIV, YFV and SINV	*In vivo* and cell line	Disrupted viral envelope and blocked viral infection	Yu et al. (2022)
*Ae. aegypti*	*Wolbachia*	*Dirofilaria immitis*	By engineered *Asaia* bacteria	inhibited the development of the heartworm parasite	Epis et al. (2020)
*Ae. albopictus*	*Wolbachia*	ZIKV	*Ae. albopictus* C3/C6 cell lines	Interfere with Zika virus replication by hijacking cholesterol metabolism of the cells	Edwards et al. (2023)
*Ae. aegypti*	*Proteus* sp. *Prpsp_P* and *Paenibacillus* sp. *Pnsp_P t*	DENV	Mosquito feeding	Decrease susceptibility to dengue infection	Ramirez et al. (2012)
*Ae. aegypti*	*E. anopheles*	ZIKV, DENV, CHIKV	Mosquito feeding and Vero cells	Reduce the Zika infection rate in mosquitoes and decrease the viral load in Vero cells.	Onyango et al. (2021)
*Ae. aegypti*	*Wolbachia* (*w*Mel and *w*ell)	MAYV	*In vivo* mosquito infection	Block viral infection and suppress viral titers	Dodson et al. (2024)
*Ae. aegypti*	*Wolbachia* infected (*wMel*) *Ae.aegypti*	DENV	Field and laboratory-reared mosquitoes infected with the virus	Lower the virus in saliva and extend EIP	Carrington et al. (2018)
*Ae. aegypti*	*Enterobacter cloacae*	DENV	*Aag2* cell line	Inhibit Dengue viral load	Barletta et al. (2020)
*Ae. aegypti*	*Wolbachia*	DENV	Analysis of Pelo protein in mosquitoes during *Wolbachia* infection	*Wolbachia* suppresses pelo protein and inhibits DENV replication	Asad et al. (2018)
*Ae. aegypti*	*Wolbachia* (NC*-w*Mel)	ZIKV, DENV and CHIKV	*In vivo,* a mosquito with (NC*-w*Mel) fed virus-infected blood	Reduced susceptibility to infection, Lack of transmission	Pocquet et al. (2021)
*Ae. aegypti*	*Lysinibacillus*	ZIKV	Forced feeding of mosquito	Decrease viral copies in the head and thorax	Do Nascimento et al. (2022)
*Aedes* and *Anopheles*	*Serratia* AS1	ZIKV *Plasmodium*	Mosquito harbored *Serratia* AS1	Inhibit infection of ZIKV and Plasmodium	Hu et al. (2025)

### 3.5 Role of mosquito’s guts microbiota in vector control

Eight articles addressed the use of mosquito microbiota in strategies for vector control. Applications of microbial-based approaches suppress the *Aedes* mosquito population. For example, an independent evaluation of *Wolbachia*-infected male (WIM) mosquito releases in Harris county, Texas, showed that CI induced by *Wolbachia* significantly reduced *Ae. aegypti* populations by over 90%. Similarly, large-scale field releases of *Ae. aegypti* mosquitoes infected with the *w*Mel strain of *Wolbachia* have led to the stable establishment of the bacterium in local mosquito populations, with a consistent prevalence of over 60% (Lozano et al., 2022).

Similarly, large-scale field releases of *Ae. aegypti* mosquitoes infected with the *w*Mel strain have led to the stable establishment of the bacterium in local mosquito populations, with a consistent prevalence of over 60%. Due to the large-scale establishment of *Wolbachia*, the incidence of dengue has been reduced (Velez et al., 2023), resulting in a 38% decrease in dengue cases and a 10% reduction in chikungunya cases (Ribeiro Dos Santos et al., 2022).

Other studies have also reported that introgression, which involves crosses between wild *Wolbachia*-infected *Ae. albopictus* males (carrying the wild *w*Pip strain) and naturally infected *w*AlbA/B females lead to complete bidirectional CI, as shown by 0% egg hatch rates. The life history traits in these wild-*w*Pip crosses were similar to those observed in laboratory crosses between lab-*w*Pip males and wild *w*AlbA/B females (Lejarre et al., 2025). Similarly, the presence of *Wolbachia* strain *w*MelM in female *Ae. aegypti* triggers fitness costs that disrupt egg retention and prevent oviposition (Ross et al., 2025). Introgression of the genetic background from a wild population into a *Wolbachia*-infected line capable of producing incompatible males (Cholvi et al., 2024).

A pilot study conducted in southern Mexico tested the integration of the Sterile Insect Technique (SIT) and the Incompatible Insect Technique (IIT) using *w*AlbB-infected *Ae. aegypti* males. These mosquitoes were mass-reared, irradiated for sterilization, and released in urban areas. After release rates resumed at the five-month mark, the intervention led to an 88.4–89.4% reduction in indoor *Ae. aegypti* presence and an overall population suppression rate ranging from 50 to 75.2% (Martín-Park et al., 2022). Similarly, combined use of IIT and SIT through the mass release of male *Ae. albopictus* mosquitoes resulted in a 62% decrease in larval abundance and a 65% decrease in adult populations over the course of a year (Zheng et al., 2019).

*Wolbachia*-based vector control has shown great promise in reducing arbovirus transmission and mosquito populations. Field releases in endemic areas have significantly decreased disease incidence. However, large-scale, sustainable implementation requires coordinated multidisciplinary collaboration, standardized methodologies, and long-term ecological monitoring to adapt to variable field conditions and maintain success (O’Neill et al., 2019; Nazni et al., 2019).

Beyond *Wolbachia*-based interventions, some resident bacteria in mosquito influence the physiology of mosquito species; cause mortality, induce the sterility and extent mosquito development. *Chromobacterium* sp. (*Csp_P*), *Chromobacterium* sp. *Panama* (*Csp_P*), isolated from field-derived *Ae. aegypti* showed strong entomopathogenic effects. Larval exposure to *Csp*_P in breeding water and adult consumption of the bacterium resulted in high mosquito mortality (Ramirez et al., 2014).

A recent study on bacteria and their metabolites isolated from *Aedes* mosquitoes demonstrated significant larvicidal activity against *Ae. aegypti* larvae. Among the most promising genera were *Bacillus* spp., *Enterobacter* spp. and *Stenotrophomonas* spp. (De Oliveira et al., 2024). Rajagopal and Ilango (2021) studied the effect of *Exiguobacterium* spp. (specifically *E. aestuarii* and *E. profundum*) on *Ae. aegypti* larvae. Exposure to different bacterial concentrations significantly prolonged larval development (from 11.41 to 14.78 days) and resulted in reduced fecundity and egg hatchability. Similarly, the *Rahnella aquatilis* isolate RAeA1, found throughout the tissues of *Ae. albopictus* was shown to impair female reproductive physiology. Inoculating adult mosquitoes with RAeA1 resulted in disrupted egg production and ovarian development due to reduced levels of ecdysteroids and vitellogenin hormones, which are essential for successful reproduction (Gu et al., 2025).

The gut microbiota of *Aedes* mosquitoes has been explored for its potential to control arbovirus through Para-transgenesis, which involves the genetic engineering of symbiotic microorganisms to express antipathogen effector molecules. Symbiotic bacterium *Serratia* AS1 has been genetically engineered to express effector molecules targeting pathogens. Mosquitoes harboring engineered Serratia demonstrated significant inhibition of *Plasmodium* and ZIKV infections in both Anopheles and Aedes mosquitoes (Hu et al., 2025; Table 2).

**Table 2:** Summaries of role of gut microbiota in vector control.

Mosquito spp.	Bacteria strain	Role in vector control	Country	Reference
*Ae. aegypti*	*w*Mel	Reduce the incidence of Dengue in an established region	Colombia, Brazil	Ribeiro Dos Santos et al. (2022) and Velez et al. (2023)
*Ae. aegypti*	*w*MelM	Disrupt egg retention, prevent mosquito oviposition	Australia	Ross et al. (2025)
*Ae. albopictus*	*w*Pip cross with naturally occurring wAlb	Bidirectional CI, and 0% egg hatching rate	France	Lejarre et al. (2025)
*Ae. aegypti*	*w*Alb(IIT) and SIT	Suppress overall population 50–72.2%	Mexico	Martín-Park et al. (2022)
*Ae. aegypti*	*Wolbachia*	Reduce population by 90% due to CI	USA	Lozano et al. (2022)
*Ae. aegypti*	*Exibguobacteria* spp.	Prolonged larval development and reduced fecundity and egg hatchability	India	Rajagopal and Ilango (2021)
*Ae. albopictus*	*Rahnella qualities* (RAeA1)	Disrupt ovarian development of females and disrupt egg production	China	Gu et al. (2025)
*Ae. aegypti* and *Ae. albopictus*	*Chromobacterium* spp.	Larvicidal activity	USA	Ramirez et al. (2014)

## 4 Discussion

In response to the challenges of vector-borne disease and the rapid development of insecticide resistance, integrated mosquito management (IMM) strategies have become increasingly important. IMM advocates for a multifaceted approach that combines chemical, biological, and environmental tools to reduce mosquito populations sustainably. Among biological control methods, bacterial larvicides like *Bti* and *Lysinibacillus sphaericus* are widely used (CDC, 2024). These bioinsecticides target larvae specifically, leaving a minimal impact on non-target organisms.

Additionally, the WHO recommends the use of symbiotic bacteria, such as *Wolbachia* and other microorganisms, to reduce the transmission of arboviral pathogens by interfering with viral replication in mosquito vectors (World Health Organization, 2016). Meanwhile, due to growing scientific interest in targeting the mosquito gut microbiota as a novel approach to control arboviral disease, this emphasized the potential of symbiotic gut bacteria in *Aedes* mosquitoes as a novel tool for inhibiting pathogen transmission and enhancing vector control.

The gut microbiota also plays a crucial role in mosquito immunity and resistance to pathogens. The presence of bacteria in the midgut can antagonize infectious agents, such as DENV and *Plasmodium*, acting as a negative factor in the vectorial competence of the mosquito (Onyango et al., 2021; Pocquet et al., 2021). Additionally, gut bacteria are involved in regulating reactive oxygen species (ROS) levels, which are essential for controlling pathogen growth and maintaining mosquito resistance to infections (Cirimotich et al., 2011b). In *Anopheles* mosquito bacteria like *Enterobacter* have been shown to enhance ROS production and reduce *plasmodium* survival in the midgut (Dennison et al., 2016).

The interaction between pathogenic and non-pathogenic microorganism started in the early stage of mosquito development by modulating the basal level of immune gene expression associated with immune response, tissue homeostasis, gut physiology, and metabolism. This microbiota-induced gene expression leads to a more rapid and robust immune response upon pathogen challenge. In *Drosophila melanogaster*, commensal bacteria upregulate antimicrobial peptide genes via the Imd pathway, enhancing resistance to subsequent infections (Broderick and Lemaitre, 2012). Similarly, in *Aedes* mosquito symbiotic bacteria’s elevated expression of several immune marker genes, including the Toll pathway related genes and modulating DENV infection (Xi et al., 2008).

Additionally, microbial interactions within the gut microbiome of *Aedes* mosquitoes are complex and involve mechanisms that enable them to evade mosquito immune responses. For example, the gut microbiome in mosquitoes utilizes C-type lectins (mosGCTLs) to counteract the bactericidal activity of antimicrobial peptides (AMPs) (Pang et al., 2016). This mechanism enables the microbiome to maintain homeostasis and colonize the mosquito’s gut successfully. Similarly, oral ingestion of bacteria triggers a robust immune response, notably antimicrobial peptides, to combat the bacteria (Lhocine et al., 2008). This suggests that interactions between the mosquito immune system and symbiotic bacteria can enhance immune priming, thereby strengthening the mosquito’s immune response against subsequent infections.

Even though the application of symbiotic bacteria for blocking pathogen transmission and suppressing mosquito populations has shown effectiveness under laboratory conditions, its implementation in field settings remains limited. One of the key issues is that bacterial communities are not static; they vary significantly across mosquito species, life stages, environmental conditions, host genome and sex, and dietary regimes (Guégan et al., 2018).

For example, the source of blood meal and mixed blood feeding influence gut bacterial community composition in mosquitoes, potentially affecting pathogen acquisition and transmission (Muturi et al., 2021b). In *Anopheles* mosquitoes difference in larval diet affects causes a change in the abundance of midgut *Enterobacteriaceae* influencing the prevalence and intensity of *P. berghei* in adults (Linenberg et al., 2016). Blood meal increases bacteria’s antioxidant activity by disturbing the compositional harmony of the consortium; this dysbiosis of microbial community may increase mosquito permissiveness for pathogenic infection.

The developmental transition from larvae to adults involves substantial remodeling of the gut and its microbiota. During the larval stage, mosquitoes develop in aquatic environments, where they acquire a diverse range of environmental bacteria. Variations in water temperature, pH, oxygen availability, and other physicochemical properties across different aquatic habitats significantly influence microbial growth and, consequently, shape the larval gut microbiota leads to ecological unpredictability in vector control (Fu et al., 2023).

Furthermore, larval exposure to different bacterial communities can result variation in adult gut microbiota, immune responses, and pathogen transmission (Dickson et al., 2017). Transient microbes present in the larval aquatic environment can be carried over to the adult stage and influence mosquito vector competence. Mosquitoes reared in environmental water containing a diverse microbial community exhibit reduced competence for Zika virus (ZIKV) transmission compared to those reared in laboratory water with limited microbial diversity (Louie and Coffey, 2021).

However, during pupation, the gut undergoes physiological renewal, including the elimination of existing microbial content via the mechanism and the replacement of the larval gut epithelium. Despite this turnover, some bacteria are retained and transmitted transstadial, contributing to the adult microbiota (Fu et al., 2023; Alfano et al., 2019). The instability of microbiota across mosquito development reduces the predictability and reproducibility of microbiota-based vector control strategies in field.

Gut symbionts also present a promising platform for delivering anti-pathogenic effectors through genetic engineering to reduce disease transmission. This method is both cost-effective and scalable, as these engineered symbionts can stably colonize various mosquito vector species and be sustained within mosquito populations through vertical, horizontal, and transstadial transmission, thereby minimizing the need for repeated reintroduction (Ratcliffe et al., 2022).

Even though genetically engineered symbionts hold great promise for targeting arbovirus and *Plasmodium* transmission and for suppressing mosquito populations, several challenges must be addressed before this approach can be widely implemented in the field. These challenges include fitness costs and genetic instability, ecological risks, horizontal gene transfer and non-target effects, as well as regulatory, ethical, and social concerns (Ratcliffe et al., 2022). To overcome these obstacles and responsibly release genetically modified mosquitoes, a multidisciplinary risk assessment, strong community engagement, and adaptive management strategies are essential to ensure sustainability and public acceptance.

Beyond pathogen suppression, gut microbiota also influences insecticide resistance. Symbiotic bacteria, such as *Serratia oryzae*, can enhance resistance to deltamethrin in *Ae. albopictus* by upregulating metabolic detoxification genes (Wang et al., 2025). This dual role, supporting both detoxification and immune defense, highlights the need to better understand microbial contributions to resistance mechanisms and their implications for control strategies. Additionally, studying the composition and functional mechanisms of the microbial community to insecticide resistance will be crucial for identifying microbial markers that could complement existing vector surveillance tools (Mantzoukas and Eliopoulos, 2020).

## 5 Conclusion

This review provides an overview of the complex and dynamic relationship between the gut microbiota of *Aedes* mosquitoes and the transmission of arboviral diseases such as Dengue, Zika, and Chikungunya. It also highlights the urgent need for innovative and sustainable vector control strategies. Different symbiotic bacteria species and strains that are taxonomically affiliated with core phyla, including Proteobacteria, Firmicutes, Actinobacteria, and Bacteroidetes, have been isolated from the gut of *Aedes* mosquitoes play pivotal roles in modulating vector competence. Understanding the factors shaping mosquito gut microbiota is the main point to developing innovative vector control strategies. Since gut bacteria influence pathogen transmission, manipulating these microbes could reduce vector competence or boost mosquito resistance. Continued research on host-pathogen interactions is vital for advancing next-generation public health vector control tools. Continued research into the mechanisms by which gut microbes interact with both their hosts and pathogens is essential for developing next-generation tools for vector control and public health. In addition to bacterial-based therapies, entomopathogenic fungi like *Beauveria bassiana* and *Metarhizium anisopliae* have shown promise in lowering mosquito populations and upsetting the balance of gut microbes, which reduces vector fitness and viral susceptibility. Further research is needed using a biomolecular approach to detect the role of gut microbes, such as viruses and fungi, as well as the mechanisms that inhibit the role of pathogenic microbes, as well as the mechanisms of competition and dominance between germs in the mosquito body, which can be the basis for vector control.

Moreover, symbiotic bacteria like *Wolbachia* have shown great promise in large-scale vector control by reducing arbovirus transmission and mosquito populations. Field releases of *Wolbachia*-infected mosquitoes have already led to significant declines in disease incidence in endemic areas. Although the reviewed studies offer compelling insights, translating microbiome-based research into scalable public health interventions requires further multidisciplinary collaboration. There is still a significant knowledge gap regarding the dynamics of microbiota in natural environments, especially when field conditions and ecological diversity are present. Long-term monitoring, evaluation of non-target impacts, and standardized microbiome manipulation techniques are necessary to further this strategy. The effective integration of mosquito gut microbiota into public health practice requires multidisciplinary research to inform interventions and continuous field evaluation within vector control programs.

Further research is needed to elucidate the mechanisms by which microbiota influences pathogen transmission fully and to explore potential applications in mosquito control efforts.
