# Supplementary material for: Microbial gatekeepers: midgut bacteria in Aedes mosquitoes as modulators of arboviral transmission and targets for sustainable vector control
Source: Front Microbiol. 2025 Sep 9;16:1656709. doi: 10.3389/fmicb.2025.1656709 (PMC12477724; doi:10.3389/fmicb.2025.1656709)
Supplement: Supplementary file 1 [file Table_1.DOCX]

Table 1: Summary of composition of Aedes mosquito gut microbiota in phylum and genera level Affiliation

| **Mosquito species** | **Source/ Origin semple** | **Bacterial Phylum** | **Bacteria Genera** | **Location** | **Procedure of identification** | **Reference** |
| --- | --- | --- | --- | --- | --- | --- |
| *Ae. aegypti* | Laboratory colonies | Proteobacteria  Acitenobacteria | *Aeromonas, Leucobacter, Ralstonia Serratia, Enterobacter* | USA | Amplification of the V3-V4 region of the 16S rRNA gene with | (Muturi, Njoroge, *et al*. 2021) |
| *Ae. albopictus* | Field collected and laboratory colonies | Proteobacteria  Firmicutes  Acitenobacteria  Bacteroidetes | *Enterobacter, Pseudomonas, Wolbachia, Vibro, Sphingomonas, Holomonas, Methylobacterium, Hydrocarboniphga, Rahnella, cupriaviadus, Bdellovibro*  *Bacillus, Clostridium, Streptococcus, Raynella*  *Propionbacterium, Pedobacter,* | Brazil | Amplification of the V3-V4 region of the 16S rRNA gene | (Baltar *et al.* 2023) |
| *Ae. albopictus* | Field collected | Proteobacteria  Bacteroidetes | *Sphingobium, Novosphingobium, Sphingomonas, Dysgonomonas, Aeromonas, Wolbachia* | France & Vietnam | Amplification of V5–V6 regions of the 16S rRNA gene | (Minard *et al*. 2015) |
| *Ae. aegypti* | Field collected | Proteobacteria Firmicutes  Tenericutes  Bacteroidetes Actinobacteria Cyanobacteria Spirochaetes and Fusobacteria | *Bacillus, Chryseobacterium, Enterobacter, Klebsiella and Serratia* | USA | reduced-representation genome sequencing (ddRAD-seq | (Pascar, *et al*, 2023) |
| *Ae. aegypti* | Field collected | Proteobacteria  Firmicutes  Acitenobacteria | *Acinetobacter, Bacillus, Brevundimonas, Corynebacterium, Delftia, Massilia, Micrococcus,* and *Stenotrophomonas* | USA | Amplification of V4 regions of the 16S rRNA gene | (Caragata *et al.* 2022) |
| *Ae. aegypti* | Laboratory colonies | Proteobacteria  Acitenobacteria  Bacteroidetes | *Asaia, Elizabethkingia, Delftia, Acenitobacter, Bifidobacterium,psedomonas, vibro, Microbacterium, Massilia* | UK (Liverpool) | Amplification of V4 regions of the 16S rRNA gene | (Brettell *et al.* 2025b) |
| *Ae. albopictus* | Field collected | Proteobacteria  Firmicutes | *Serratia, Aeromonas, Delftia, Paracoccus, Planomicrobium,* and *Asaia* | Iran | Culture dependent and 16S rRNA gene (V1–V9 regions | (Darbandsari *et al.* 2025) |
| *Ae. albopictus* | Laboratory colonies | Bacteroidetes and Proteobacteria | *Elizabethkingia, Pseudomonas, Sphingomonas,* and *Wolbachia* | USA | Amplification of the V3-V4 region of the 16S rRNA gene | (Onyango *et al.* 2024) |
| *Ae. aegypti* & *Ae. albopictus* | Field collected | Proteobacteria  Firmicutes  Spirochetota | *Aeromonas, Wolbachia, Clostridium innocuum group, Coprobacillus, Blautia, Parasutterella, Akkermansia, Bifidobacterium, Castellaniella, Morganella, Klebsiella, Escherichia-Shigella, and Clostridioides* | Northeastern Thailand | Amplification of the V3-V4 region of the 16S rRNA gene | (Rodpai *et al*. 2023) |
| *Ae. albopictus* | Field collected | Proteobacteria  Firmicutes  Actinobacter  Bacteroidetes and Tenericutes | *Wolbachia, Xanthomonas, Zymobacter, unidentified bacteria Xenophilus and Acinetobacter* | Malaysia | Amplification of the V3-V4 region of the 16S rRNA gene | (Lee *et al.,* 2020) |
| *Ae. albopictus* | Field collected | Proteobacteria  Firmicutes  Actinobacter  Bacteroidetes | *Pseudomonas, Dysgonomonas, Limnobacter* | Italy, France and Vietnam | Amplification of the V5-V6 region of the 16S rRNA gene | (Rosso *et al.* 2018) |
| *Ae. albopictus* | Laboratory colonies | Proteobacteria  Firmicutes | *Aquabacterium, Methylophilus, Nevskia, Solimonas Sphingomonas,*  *Asaia, Dechloromonas, Aerococcus, Bacillus, Brevundimonas, Pseudomonas, Rhizobium, Stenotrophomonas, Acinetobacter, Pelomonas, Ralstonia,* and *Wolbachia* | Spain and São Tomé | Amplification of the V3-V4 region of the 16S rRNA gene | (Melo *et al.* 2024) |
| *Ae. aegypti & Ae. albopictus* | Field collected | Proteobacteria  Actinobacter  Firmicutes  Bacteroidetes  Ascomycota(fungus) | *Zymobacter, Bacillus, Wolbachia, Enterobacter, Ornithimicrobium and fungus Smittium, Conidiobolus, Metarhizium, Tolypocladium,*  *Pythium* and *Beauveria* | Mexico | Amplification of the V3-V4 region of the 16S rRNA gene | (Hernández *et al.* 2024) |
| *Ae. aegypti* | Laboratory colonies | Proteobacteria Bacteroidetes  Firmicutes  Actinobacter  Aquifcota  Chlorofexi Cyanobacteria Deinococcota Desulfobacterota Myxococcota  Verrucomicrobiota | *Enterobacter, Pseudomonas, Enterobacteriaceae, Raoultella, Asaia, Gluconobacter, Chryseobacterium, Shewanella,* and *Acinetobacter* | Thailand | Amplification of the V3-V4 region of the 16S rRNA gene | (Siriyasatien *et al.* 2024) |
| *Ae. albopictus* | Laboratory colonies | Proteobacteria  Firmicutes | *Geobacillus, Wolbachia, Tanticharoenia, Enterobacter, Klebsiella,* and *Escherichia coli* | China  Mexico  Italy | Amplification of the V3-V4 region of the 16S rRNA gene | (Bahrami *et* *al.* 2024) |
| *Ae. albopictus* | Field collected | Proteobacteria  Bacteroidetes  Firmicutes Acinetobacter | *Wolbachia, Acinetobacter, Providencia,* and *Morganella* | South Korea | Amplification of the V4 region of the 16S rRNA gene | (Akintola and Hwang 2024) |
| *Ae. albopictus* | Field collected | Proteobacteria Firmicutes Actinobacteria Bacteroidetes | *Acinetobacter, Microbacterium,*  *Micrococcus, Stenotrophomonas, Klebsiella, Pseudomonas, Enterobacter, Aeromonas, Clostridium* and *Bacillus* | Northeastern India. | Amplification of the V1-V9 region of the 16S rRNA gene | (Yadav *et al.* 2016) |
| *Ae. aegypti* | Field collected | Proteobacteria  Firmicutes  Bacteroidetes  Actinobacteria | *Staphylococcus, Serratia, Rhodobacter, Raoultella Pseudomonas, Limnobacter*  *Leclercia, Elizabethkingia*  *Dietzia, Curvibacter*  *Corynebacterium, Caulobacter*  *Brevundimonas, Bacillus* and  *Acinetobacter* | India | Amplification of the V2, V3,V4,V6-V9 region of the 16S rRNA gene | (Sarma *et al.* 2022) |
| *Ae, albopictus* | Field collected | Proteobacteria  Firmicutes  Actinobacteria  Deinococcus Thermus | *Curtobacterium flaccumfaciens, Microbacterium, Arthrobacter, Kocuria, Streptomyces, Bacillus, Staphylococcus, Haematobacter massiliensis, Enterobacter, Klebsiella oxytoca, Acinetobacter and Pantoea. Asaia, Citrobacter freundii and Sphingomonas Arseniococcus bolidensis, Cellulosimicrobium, Deinococcus, Leucobacter, Planococcus, Pseudomonas, Skermanella aerolata,* and *Xanthomonas* | Madagascar | Culture dependent and Amplification of the V1-V9 region of the 16S rRNA gene | (Valiente Moro *et al.* 2013) |
| *Aedes* and *culex spp* | Field collected | Proteobacteria | *Asaia, Pseudomonas, Delfita, Wolbachia, Serratia, Pantoea Enterobacter,* and *Rahodobacter*, | Italy, Cameron | Amplification of the V3-V4 region of the 16S rRNA gene | (Ilbeigi Khamseh Nejad *et al.* 2024) |
| *Ae. albpictus & Ae. aegepti* | Laboratory colonies | Proteobacteria Bacteroidetes  Firmicutes Acitenobacteria  Cyanobacteria | *Methylotenera, Asaia, Enterobacter, Wolbachia, Bacillus, Rastonia, Sphigomonas,* and  *Leptothnx* | Southern China | Amplification of the V3-V4 region of the 16S rRNA gene | (Lin *et al.* 2021) |
| *Ae. albpictus* | Field collected and laboratory colonies | Proteobacteria  Firmicutes Actinobacter | *Bacillus subtilis,*  *Staphylococcus*  *Haemolyticus, Staphylococcus hominis, Serratia marcescens, Enterobacter cloacae, Micrococcus luteus,* and *Providencia rettgeri,* | Thailand | Amplification of the V1-V9 region of the 16S rRNA gene | (Tuanudom *et al.* 2021) |
| *Ae. albopictus* and *Ae. aegypti* | Field collected | Proteobacteria  Bacteroidetes  Firmicutes  Acitenobacteria | *Enterobacter cloacae, Klebsiella michiganensis, Pseudomonas monteilii, Bacillus aryabhattai, Lysinibacillus fusiformis,* and *Staphylococcus hominis* | India | Culture dependent and Amplification of the V3-V4 region of the 16S rRNA gene | (Yadav *et al*. 2015) |
| *Ae. aegypti* | Laboratory colonies | Proteobacteria  Firmicutes  Acitenobacteria | *Asaia, Bacillus, Enterobacter, Enterococcus, Klebsiella* and *Serratia* | Brazil | Culture-dependent Amplification of the V1-V9 region of the 16S rRNA gene | (Gusmão *et al.* 2010) |
| *Ae, aegypti* | Laboratory colonies | Proteobacteria  Firmicutes | *Bacillus, Elizabethkingia, Enterococcus, Klebsiella, Pantoea, Serratia,* and *Sphingomonas* | Sweden | Culture dependent and Amplification of the V6-V8 region of the 16S rRNA gene | (Terenius *et al*. 2012) |

*Table 2: Summary of composition of Aedes mosquito gut microbiota in phylum and genera level Affiliation*

| **Reference** | **Mosquito Stage /Sample type** | **Laboratory Procedure** | **Method Used** | **Major Finding** |
| --- | --- | --- | --- | --- |
| (MacLeod, *et* *al.* 2021) | Larvae, adults | Rearing with different diets, 16S rRNA sequencing | Comparative microbiota profiling | Larval diet abundance Influences size and composition of the midgut microbiota of *Aedes aegypti* mosquitoes |
| (Linenberg, *et al.*,2016) | Larvae | Diet control, 16S rRNA sequencing | Microbiota comparison under diet | Larval diet affects mosquito development and significant increase a certain bacterium |
| (Martinson and Strand 2021) | Larvae | Bacterial inoculation, nutrient manipulation | Nutritional-microbial co-treatment | Larval diet and bacterial supplementation shaped microbiota |
| (Yadav *et al.* 2016) | Larvae, sugar-fed & blood-fed adults | 16S sequencing, species isolation | Community profiling across variables | Microbial composition varied with stage, sex, and feeding |
| (Scolari *et al*. 2021) | Breeding water, larvae, adults | Water sampling, microbiota sequencing | Environmental-mosquito microbial overlap | Environmental water shared bacterial taxa with larvae and adults |
| (Juma *et al.* 2021) | Larvae, water samples | Site comparison, microbial DNA sequencing | Geographically stratified sampling | larval environment strongly influences the bacterial communities |
| (Rodpai *et al. 2023)* | Larvae, adults | Whole-body microbiota sequencing | Developmental stage comparison | Transstadial transmission observed; reduced load in adults |
| (Sarma *et al.* 2022) | Adult females (fed/unfed) | Feeding experiments, gut dissection, 16S profiling | Blood-fed vs unfed comparison | Blood feeding shifts gut microbiota composition |
| (Muturi *et al.* 2018) | Adult females (fed with different hosts) | Controlled feeding, midgut analysis | Host-blood source effect | Blood source affects microbial profiles |
| (Salgado *et al.* 2024) | Blood-fed adult females | 16S sequencing after digestion | Temporal microbiota shifts after feeding | Microbial diversity reduced post blood-feeding; dominated by *Enterobacterales* |
| (LaReau *et al.* 2023) | Adults | Germ-free rearing, environmental bacterial inoculation | Axenic rearing with inoculation | Axenic vs environment-colonized mosquitoes show different gut profiles |
| (Frankel-Bricker *et al.* 2020) | Larvae, adults | Fungal exposure, sequencing | Cross-stage microbiota tracking | Fungal colonization in larvae affects adult gut diversity |
| (Yin *et al.* 2025) | Larvae, pupae, adults | Experimental inoculation | Microbial manipulation | Inoculation with *E. coli*, *S. aureus*, *B. bassiana* alters microbiota composition |
| (G. Wei et al. 2017) | Adults | Infection with spores, sequencing | Fungal infection study | *B. bassiana* infection causes gut dysbiosis and mortality |
| (Balaji, *et al.*, 2021) | Adults | Infection and gut profiling | *Wolbachia*-infection impact study | *Wolbachia* alters microbiota whereas *Serratia* enriched |
| (Zhao *et al*., 2022) | Adults (infected) | Viral challenge, qPCR and sequencing | Virus-microbiota interaction | DENV infection shifts microbiota and reduces load |
| (Arévalo-Cortés *et al.,* 2022) | Adults (resistant/infected) | Exposure assay, microbiota profiling | Dual stressor study | Insecticide-resistant and ZIKV-infected mosquitoes show altered gut profiles |
| (X. Wei *et al.*, 2023) | Adults | Exposure to insecticides, microbiota analysis | Insecticide exposure | Pyrethroid exposure alters bacterial diversity |
| (Muturi *et al.* 2021; Viafara-Campo *et al.* 2025) | Larvae, adults | Resistance assay, gut profiling | Resistance phenotype comparison | Resistant vs susceptible mosquitoes have different microbiota |
| (Qing *et al.* 2020) | Larvae, adults | Exposure to antibiotics, sequencing | Antibiotic treatment | Ampicillin causes dysbiosis, esp. in adult females |
| (Garcia--Van Smévoorde *et al.*, 2024) | Adults | Antibiotic feeding, DENV challenge | Combined stressor study | Antibiotics + DENV alter gut microbiota composition |
| (Guégan *et al.*, 2018) | Larvae, adults | Antibiotic rearing | Larval antibiotic exposure | Antibiotics in larvae reduce Elizabethkingia and increase *Wolbachia* |
| (Antonelli *et al*., 2024) | Larvae, adults | PAH chronic exposure, sequencing | Pollutant exposure | PAH pollution shifts gut microbiota and enriches PAH degraders |
| (Hegde *et al.,* 2019) | Larvae, adults | CRISPR/Cas9 editing, colonization assays | CRISPR-mediated gene deletion | *ompA* gene knockout reduces colonization |
| (Minard *et al.* 2015) | Adults | Field sampling, diversity analysis | Geographic comparison | Invasive mosquitoes show lower microbial diversity |
| (Brettell *et al.*, 2025) | Eggs, larvae, adults | Controlled insectary conditions | Environmental rearing condition | Different insectaries yield different microbiota from same eggs |
| (Melo *et al.* 2024) | Adults | DNA sequencing from different regions | Cross-region microbiota comparison | Geographic strains of *Ae. albopictus* share core but differ in specific taxa |
| (Baltar *et al.* 2023) | Adults | Field vs lab rearing, seasonal sampling | Environment and season comparison | Lab vs field mosquitoes differ; seasonal variation observed |
